# WHO assessment of the preclinical antifungal pipeline: evaluating innovation and preparedness in the face of emerging fungal threats

**DOI:** 10.1016/j.lanmic.2025.101331

**Published:** 2026-08

**Authors:** Valeria Gigante, Richard A Alm, Tamarie Rocke, Daniela Melchiorri, Alexandra M Cameron

**Affiliations:** a Antimicrobial Resistance Department, WHO, Geneva, Switzerland; b Department of Physiology and Pharmacology, Sapienza University of Rome, Rome, Italy

## Abstract

Life-threatening invasive and mucosal fungal infections are a major, increasingly complex global health problem. Among all population groups, individuals who are immunocompromised face the greatest risk of contracting such infections, and the size of these global populations is growing at a swift pace. Climate change also affects the dynamics of fungal infections. Commercially available antifungal drugs often have a narrow-spectrum activity and below-optimal safety profile. Drug resistance to antifungals is also rising, and the use of fungicides in agriculture plays a role in resistance development. In this Review, we aim to provide an overview of the current preclinical research and development landscape of antifungal agents and to critically assess the extent to which current efforts address fungal priority pathogens according to the WHO priority list. We identified 22 programmes in the preclinical pipeline, including 13 for novel agents, four exploring new routes of administration, two investigating new formulations of existing antifungals, and three repurposed monoclonal antibody programmes. These preclinical programmes are at the initial stages (with nine [40·9%] of 22 programmes in the earliest preclinical stage) and insufficiently mature to enter robust clinical developmental stages, requiring comprehensive safety characterisation before advancement.

Thus, there is a crucial need to develop novel antifungal agents with improved potency, broad-spectrum activity, and enhanced safety profiles and pharmacological features.

## Introduction

Fungal infections pose a major yet under-recognised global health threat, as they are challenging to diagnose and becoming increasingly prevalent and resistant to existing treatments. The paucity of appropriate diagnostics and surveillance studies makes the accurate identification of the global burden of disease difficult. The 2024 estimates of incidence and attributable mortality indicate that there are 6·5 million invasive fungal infections (IFIs) and 3·8 million deaths caused by IFIs annually.^1^^,^^2^ As the prevalence increases after natural disasters, such as floods, hurricanes, and wildfires,^3^^,^^4^ global climate change is also likely to increase the prevalence of IFIs. Both long-term climate-driven shifts and short-term extreme events can contribute to the adaptation of fungal pathogens, expansion of their geographical distribution, increased incidence, enhanced resistance mechanisms, and exacerbation of disease burden.

Disparities in social determinants of health increase the risk of IFIs in some individuals and populations, particularly those in occupations with heightened exposure to pathogenic fungi (eg, agricultural and construction workers).^5^ Conflicts, poverty, environmental disasters, and population displacement also contribute to the widespread expansion of IFIs.^6^ IFIs represent a serious and mounting public health concern aggravated by additional external factors, such as the increase in ageing populations with weakened immune systems and fungicide use in agriculture.

The upsurge^1^ in the burden of IFIs is also intricately linked to the globally rising number of individuals who are immunocompromised with weakened natural defences due to underlying conditions such as cancer, HIV/AIDS, organ transplantation, diabetes, or the use of immunosuppressive therapies.^3^ These individuals are at higher risk of developing life-threatening fungal infections than the general population, and survival depends on timely diagnosis and prompt initiation of effective antifungal therapy. However, both diagnosis and treatment of IFIs are challenging owing to the following reasons.

IFIs might present atypical clinical symptoms that overlap with those of other common conditions. Co-infection and comorbidity frequently pose further obstacles to diagnosis.^5^^,^^7^ Viral infections such as influenza and COVID-19 are now well recognised risk factors for invasive aspergillosis, including influenza-associated pulmonary aspergillosis and COVID-19-associated pulmonary aspergillosis, which occur in individuals not conventionally considered immunocompromised.^3^^,^^5^ Only a few classes of antifungal agents (ie, azoles, echinocandins, and polyenes) are currently available to treat severe IFIs. However, most of them have limitations in their spectrum of activity, efficacy, and safety profile. In addition, the spread of drug resistance among fungi complicates therapy.^1^

Fungi adapt to diverse environments, including in the presence of antifungals, owing to their genomic plasticity enabled by varying genome sizes and frequent rearrangements within species. Fungi can gain extra chromosomes that carry resistance-causing genes, lose heterozygosity to retain only drug-resistance alleles (as seen in *Candida albicans*), or increase the copy number of specific resistance genes. Genomic plasticity also encompasses mechanisms such as point mutations, gene deletions, and mitochondrial loss, all of which can contribute to antifungal resistance. However, resistance is not solely driven by genome architecture; epigenetic regulation, metabolic adaptation, and stress response pathways also play crucial roles in enabling fungal survival under antifungal pressure. These adaptations make fungal infections difficult to treat. Antifungal agents used in agriculture promote the development of resistance, highlighting the need for a One Health approach to address antifungal resistance.^2^ Recent additions to the antifungal arsenal, such as ibrexafungerp—a triterpenoid antifungal with a novel mechanism of action targeting the fungal cell wall—in 2021, offer promising alternatives for IFIs, particularly for infections caused by drug-resistant *Candida*.

In response to the escalating morbidity and mortality attributed to fungal infections, in 2022, WHO published the first fungal priority pathogens list (FPPL)^7^ based on public health importance, treatment challenges, and emerging resistance patterns. The FPPL was developed using a structured, evidence-based methodology to identify fungal species that pose the greatest threat to global public health. The FPPL prioritises 19 fungal pathogens, grouped into critical-priority, high-priority, and medium-priority categories on the basis of factors such as incidence, mortality, antifungal resistance, and impact on vulnerable populations, particularly in low-income and middle-income countries. The list recommends, among other interventions, sustaining investments in research and development (R&D), and innovation to address the issue of infections caused by fungal priority pathogens (FPPs). *Cryptococcus neoformans, Candidozyma auris (Candida auris), Aspergillus fumigatus,* and *C* *albicans* are classified under the critical-priority group in the FPPL. *Nakaseomyces glabratus (Candida glabrata), Histoplasma* spp, Eumycetoma causative agents, Mucorales, *Fusarium* spp, *Candida tropicalis,* and *Candida parapsilosis* are included in the high-priority group. The medium-priority group comprises *Scedosporium* spp, *Lomentospora prolificans*, *Coccidioides* spp, *Pichia kudriavzevii* (*Candida krusei*), *Cryptococcus gattii*, *Talaromyces marneffei, Pneumocystis jirovecii,* and *Paracoccidioides* spp.

The first FPPL was a landmark WHO document, which aimed to stimulate R&D for diagnostics, therapeutics, and vaccines in an area that has been historically underfunded and under-recognised.^7^ Complementing the development of the FPPL, WHO performed a global pipeline analysis in 2025 to examine the status of both the clinical and preclinical R&D of antifungal agents targeting the WHO FPPs.^2^ Together, these two WHO initiatives underscore the importance of aligning R&D investments with the most urgent fungal threats, ensuring that global health responses are proactive, equitable, and scientifically grounded.

Several innovative agents in the clinical antifungal pipeline,^2^ which are active against the fungal pathogens in the critical-priority group, are in late-stage clinical development. Two of such agents—fosmanogepix (Basilea Pharmaceuticals) and olorofim (F2G and Shionogi)—have novel mechanisms of action and belong to novel chemical classes. Four agents in phase 1 trials are undergoing safety evaluation in healthy individuals, which is often a key hurdle in antifungal development.^2^ Although some recently approved antifungal agents and other novel agents in clinical trials offer hope to physicians treating fungal infections and individuals who are infected, long-term sustainability of the pipeline is imperative to ensure the availability of appropriate medicines to treat IFIs safely and effectively. Such sustainability can be accomplished only with a preclinical pipeline with the breadth and depth to withstand attrition, while still fostering clinical development. Therefore, the preclinical pipeline should account for dropout rates and the risks and challenges associated with earlier developmental stages. In this Review, we critically evaluate the 2024 preclinical antifungal pipeline and offer a comprehensive overview of the current R&D landscape for IFIs, fostering the development of innovative and effective therapies targeting the WHO FPPs. Complementary analysis of the clinical antifungal pipeline is being addressed in a separate paper.

This Review aims at steering R&D towards the most pressing public health needs by guiding drug developers and researchers across large pharmaceutical companies, biotech start-ups, academic institutions, and product development partnerships. This Review can also serve as a resource for public and private research funders to inform long-term investment strategies and support policy makers, regulatory authorities, infectious disease experts, and clinicians in evaluating the current pipeline or forecasting future needs. Finally, this Review seeks to empower health-care providers and advocacy groups to promote interventions that are appropriate, scalable, affordable, and equitable.

## Methods

This Review focused on antifungal agents that target the WHO FPPs and are in various stages of the preclinical pipeline, from lead optimisation (post-hit expansion) to the initiation of phase 1 clinical testing. The following definitions were used for classifying candidates in the preclinical pipeline. Lead optimisation refers to the iterative process of refining hit compounds through in-vitro and in-vivo screening to generate high-quality and reproducible pharmacological, safety, and pharmacokinetic data. A preclinical candidate is a compound that has successfully passed initial toxicology assessments and shown a favourable safety profile and pharmacological activity in animal models. Compounds in the clinical trial application or investigational new drug (IND) stage should be subjected to comprehensive absorption, distribution, metabolism, and excretion studies, good laboratory practice-compliant toxicology evaluations in animal models, and formulation and manufacturing development studies to obtain regulatory approval to start phase 1 clinical trials.

We used both traditional and non-traditional approaches and considered direct-acting antifungals, indirect-acting antifungals, and repurposed agents, including those with a new proposed route of administration. The assessment did not include diagnostic, antibacterial, antiviral, antiparasitic, or antifungal agents intended for wound care or industrial or veterinary use. This Review was informed by a multistep data collection process combining a WHO open data call, desktop review, literature search, and expert consultation.

To generate the primary dataset, a WHO online data call was opened between April 22 and June 17, 2024. Through the data call, developers were invited to provide information on the following attributes: product name, programme name, or descriptor; company or institution name; country of company or institution; WHO region (African region, European region, Eastern Mediterranean region, region of the Americas, Western Pacific region, and South-East Asia region); income group; type of company or institution; size of company or institution; product development stage; product type; product primary intervention stage; mechanism of action; and activity against target pathogens. The call was disseminated across several platforms, including the WHO website,^8^ via direct communication (eg, emails and calls) with developers leveraging the Australian AMRNet^9^ and ILSE networks, social media platforms (LinkedIn and Twitter), and via communications during conferences (eg, European Society of Clinical Microbiology and Infectious Diseases Global 2024). Data were self-declared by institutions. The data were supplemented with information from Biotech companies in Europe combating antimicrobial resistance (BEAM) Alliance, which collected information from its member companies using the same WHO data-call template as that used in this Review.

To supplement the data call, we did a desktop review by drawing on publicly available sources such as institutional websites, regulatory databases (ie, US Food and Drug Administration, European Medicines Agency, Therapeutic Goods Administration, and the Medicines and Healthcare products Regulatory Agency), abstracts and posters presented during scientific conferences on infectious diseases, funding agency portals (eg, Antimicrobial Resistance Action Fund, and the Biomedical Advanced Research and Development Authority^10^), and national funding agencies (ie, the German Research Foundation, UK Research and Innovation, National Health and Medical Research Agency, US National Science Foundation, and America's Seed Fund). We searched PubMed, Embase, and Google Scholar from Jan 1, 2022, to July 31, 2024, to identify relevant publications on antifungal agents in the preclinical development stage; the search was restricted to English-language publications. The review focused on systemic antifungal candidates intended for serious acute and subacute fungal diseases caused by WHO fungal priority pathogens. Search terms included combinations of *“*antifungal agents,” “preclinical development,” “fungal priority pathogens,” “*Candida*,” “*Aspergillus*,” “*Cryptococcus*,” “novel antifungals,” “lead optimisation,” “IND-enabling studies,” and “antifungal resistance.” WHO validated the accuracy of data, when possible, through expert opinion during the WHO expert consultation dated July 8, 2024. Individual follow-up with developers was also done to gather further information, and expert opinion was sought. TR developed the data call platform and webpage and then collected the data submitted through the platform. RAA performed the desktop and literature searches and data extraction. Data were revised by RAA, and duplicates were omitted. The validated dataset is available and accessible on WHO webpage, along with a data visualisation dashboard.^11^

## Results

### Demographic analyses of programmes captured in the preclinical pipeline

The augmented global data call captured 22 preclinical programmes affiliated with 18 institutions (table).

**Table:** Products or programmes captured in the 2025 WHO analysis of antifungal agents in preclinical developmental stage mapped against the WHO critical-priority fungal pathogens

	Type of compound	Stage of development	Developer (size)∗	Preclinical company type	Country†	WHO region	Antifungal class or product type	Mode of action	Target critical FPP‡	Sought-after indications	ROA	New or repurposed product
**MCC_010182**	Traditional	Lead optimisation	Institute for Molecular Bioscience, The University of Queensland (large)	Academic institution or university	Australia	WPR	Direct acting–small molecule	Unknown	*C auris, C albicans,* and *C neoformans*	To be determined	po	NCE
**DT-3-318 series**	Traditional	Preclinical candidate	Westmead Institute for Medical Research (large)	Academic institution or university	Australia	WPR	Direct acting–small molecule	Targets central metabolism	*C albicans* and *C neoformans*	Cryptococcal meningitis	iv	NCE
**SCY-247**	Traditional	IND-enabling studies on selected candidate(s)	SCYNEXIS (small)	Public company	USA	AMR	Direct acting–small molecule	Targets cell wall biogenesis	*A fumigatus,C auris,* and *C albicans*	Invasive fungal infection	iv or po	NCE
**ATB1651 (derivatives)**	Traditional	Lead optimisation	AMTIXBIO (small)	Private company	South Korea	WPR	Direct acting–small molecule	Targets cell wall biogenesis	*A fumigatus, C auris, C albicans*, and *C neoformans*	Onychomycosis	iv, po, sc, im, inhaled, or topical	NCE
**Luliconazole**	Traditional	Lead optimisation	Amity Institute of Pharmacy (medium)	Academic institution or university	India	SEAR	Direct acting–small molecule	Targets cell wall biogenesis	*A fumigatus, C auris,* and *C albicans*	Tinea	topical	Repurposed (formulation) entity
**Next- generation antifungal antibodies**	Non-traditional	Preclinical candidate	Brigid Biologics, University of Aberdeen (large)	Academic institution or university	UK	EUR	Indirect acting–large molecule	Targets immunomodulation	*C auris* and *C albicans*	Invasive candidiasis or candidemia	iv	NBE
**NP339**	Traditional	IND-enabling studies complete	NovaBiotics (small)	Private company	UK	EUR	Direct acting–peptide	Direct membrane effect	*A fumigatus, C auris, C albicans*, and *C neoformas*	Invasive and respiratory fungal infections	iv or inhaled	NCE
**slm100**	Traditional	Lead optimisation	Selmod (micro)	Private company	Switzerland	EUR	Direct acting–small molecule	Targets cell wall biogenesis	*A fumigatus, C auris,* and *C albicans*	Invasive aspergillosis	iv or inhaled	NCE
**BSG005**	Traditional	IND submitted (ready for commencement of human testing)	Biosergen AS (micro)	Public company	Norway	EUR	Direct acting–small molecule	Targets cell wall biogenesis	*A fumigatus, C auris, C albicans*, and *C neoformas*	Invasive aspergillosis	po or inhaled	NCE (new ROA)
**OCF001**	Traditional	IND-enabling studies on selected candidate(s)	Sano Chemicals Inc. (small)	Private company	USA	AMR	Direct acting–small molecule	Targets other cellular function	*C auris, C albicans*, and *C neoformas*	Vulvovaginal candidiasis	iv or topical	NCE
**Opelconazole (PC945)**	Traditional	IND submitted (ready for commencement of human testing)	Pulmocide Ltd. (small)	Private company	UK	EUR	Direct acting–small molecule	Targets ergosterol binding, increases fungal membrane permeability	*A fumigatus, C auris, C albicans*, and *C neoformas*	Invasive aspergillosis	inhaled	NCE (new ROA)
**Olorofim (orotomides)**	Traditional	Preclinical candidate	F2G Ltd. (small)	Private company	UK	EUR	Direct acting–small molecule	Targets DNA replication or synthesis	*A fumigatus*	Invasive aspergillosis	iv or inhaled	NCE (new ROA)
**SF001**	Traditional	Preclinical candidate	Elion Therapeutics (small)	Private company	USA	AMR	Direct acting–small molecule	Direct membrane effect	*A fumigatus* and *C albicans*	Invasive aspergillosis	po	NCE (new ROA)
**Anti-*Aspergillus* CART cells; GXMR–CART cells**	Non-traditional	Preclinical candidate	University of Texas MD Anderson Cancer Center (large)	Academic institution or university	USA	AMR	Biological (Antibody-based or other cell-based) molecule	Targets immunomodulation	*A fumigatus*	Invasive aspergillosis	iv	NCE
**GMXR–CART cell product**	Non-traditional	Preclinical candidate	University of Texas MD Anderson Cancer Center (large)	Academic institution or university	USA	AMR	Biological (Antibody-based or other cell-based) molecule	Targets immunomodulation	*C neoformans*	Cryptococcosis	iv	NCE
**DectiSomes**	Non-traditional	Preclinical candidate	University of Georgia (large)	Academic institution or university	USA	AMR	Liposomes	Direct membrane effect	*A fumigatus, C auris, C albicans*, and *C neoformans*	Invasive aspergillosis	iv, po, sc, im, inhaled, or topical	Repurposed (formulation) entity
**VX01**	Non-traditional	Preclinical candidate	Vitalex Biosciences (micro)	Private company	USA	AMR	Biological (Antibody-based or other cell-based) molecule	Targets immunomodulation	Mucorales	Mucormycosis	iv	NBE
**NXT2-mab 1–17**	Non-traditional	Lead optimisation	NXT Biologics (micro)	Private company	USA	AMR	Biological (Antibody-based or other cell-based) molecule	Unknown	*A fumigatus, C auris,* and *C albicans*	Invasive aspergillosis	sc or inhaled	NBE
**DHFR**	Traditional	Lead optimisation	Kathera Bioscience (micro)	Private company	USA	AMR	Direct acting–small molecule	Targets central metabolism	*A fumigatus, C auris, C albicans*, and *C neoformans*	Multiple	iv or po	NCE
**Essential genes program 1**	Traditional	Lead optimisation	Kathera Bioscience (micro)	Private company	USA	AMR	Direct acting–small molecule	Direct membrane effect	*A fumigatus, C auris, C albicans*, and *C neoformans*	Multiple	iv or po	NCE
**Essential genes program 2**	Traditional	Lead optimisation	Kathera Bioscience (micro)	Private company	USA	AMR	Direct acting–small molecule	Targets DNA replication or synthesis	*A fumigatus, C auris, C albicans*, and *C neoformans*	Multiple	iv or po	NCE
**Broad spectrum antifungal**	Traditional	Lead optimisation	Kathera Bioscience (micro)	Private company	USA	AMR	Direct acting–small molecule	Unknown	*A fumigatus, C auris, C albicans*, and *C neoformans*	Multiple	iv or po	NCE

*A fumigatus=Aspergillus fumigatus*. AMR=region of the Americas. CAR-T=chimeric antigen receptor-positive T cells. *C albicans=Candida albicans. C auris=Candidozyma auris (Candida auris). C neoformans=Cryptococcus neoformans.* CPP=critical-priority pathogen. EUR=European region. GXMR=glucuronoxylomannan receptor. HIC=high-income country. im=intramuscular. IND=investigational new drug. iv=intravenous. LMIC=low-income and middle-income countries. NBE=new biological entity. NCE=new chemical entity. po=oral. OPP=other priority pathogen (high- and medium-priority groups). ROA=route of administration. sc=subcutaneous. SEAR=South-East Asia region. WPR=Western Pacific region.

∗ Large indicates a developer size greater than 501 employees; medium indicates a size of 51–501 employees; small indicates a size of 11–50 employees; micro indicates fewer than ten employees.

† All developers are from high-income countries except for Amity Institute of Pharmacy, which is from India, a lower-middle-income country.

‡ All products except VX01 address one or more critical priority pathogens; VX01 specifically targets Mucorales in the high-priority group. Of note, four programmes only target CPPs, whereas the remaining 17 programmes also include OPPs.

**Figure 1:**
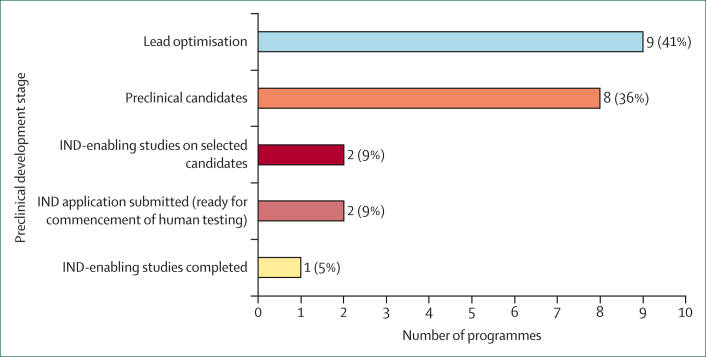
Number of products or programmes by preclinical developmental stage IND=investigational new drug.

Of these 22 preclinical antifungal programmes, nine (41%) were at the lead-optimisation stage, the earliest stage included in the analysis; compounds in eight (36%) programmes were preclinical candidates, and five (23%) were undergoing or had completed IND-enabling studies (figure 1).

**Figure 2:**
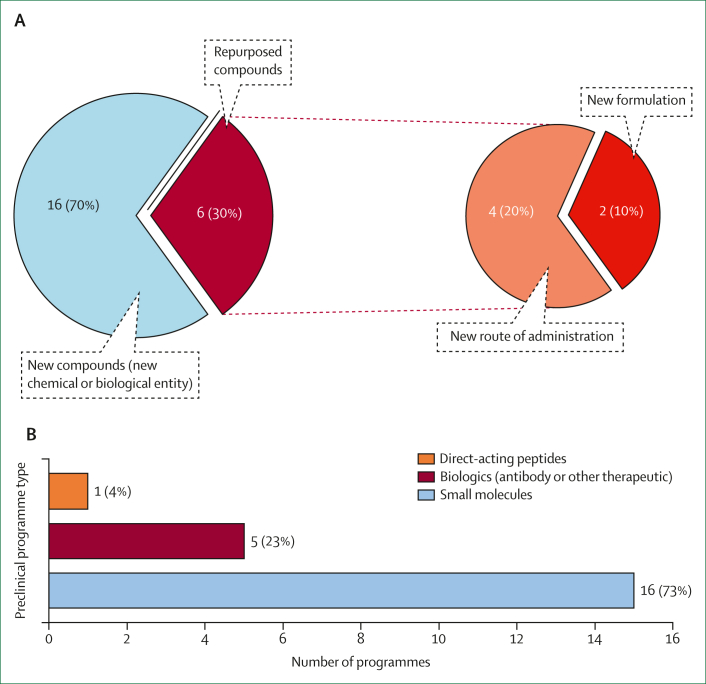
Classification of preclinical agents (A) New versus repurposed entities. (B) Classification by type of molecule or entity.

These statistics, although derived from few programmes, indicate that most preclinical candidates are at an early stage, making the preclinical pipeline insufficiently mature to generate the number of projects required to sustain a robust clinical pipeline, wherein costs and complexity of R&D further increase. Candidates from 16 (73%) of 22 programmes were classified as new agents (figure 2A). The remaining six programmes involved agents in which key performance characteristics were being altered to provide clinical differentiation, or molecules that were being repurposed to leverage potential antifungal properties. Four programmes involved the investigation of a new route of administration for a previously known active antifungal agent; a new formulation was being evaluated in two programmes. Although these products might be effective for clinical care in the short term, they substantially reduce the proportion of novel molecules being developed to address continually evolving pathogens. 17 (77%) of the 22 programmes focused on a direct-acting molecule, 16 (73%) of which involved traditional small molecules, and a peptide. Five (23%) antibody-based or cell-based biological products were also included (figure 2B).

The 22 antifungals in the preclinical pipeline were evaluated against the WHO FPPL to quantify the number of programmes in which agents targeting each of the 19 FPPs were being investigated (table).

For all FPPs, although specific agents with some proof of activity were available in the preclinical pipeline, the critical-priority pathogens had a considerably higher number of targeting products than pathogens of other groups. Nine programmes included compounds that were active against all four members of the critical-priority pathogen group; seven of these nine were new compounds and two were compounds for which a new administration route was being investigated. There was only one programme targeting a single FPP—an antibody-based programme targeting Mucorales.

### Geographical distribution of preclinical programmes

**Figure 3:**
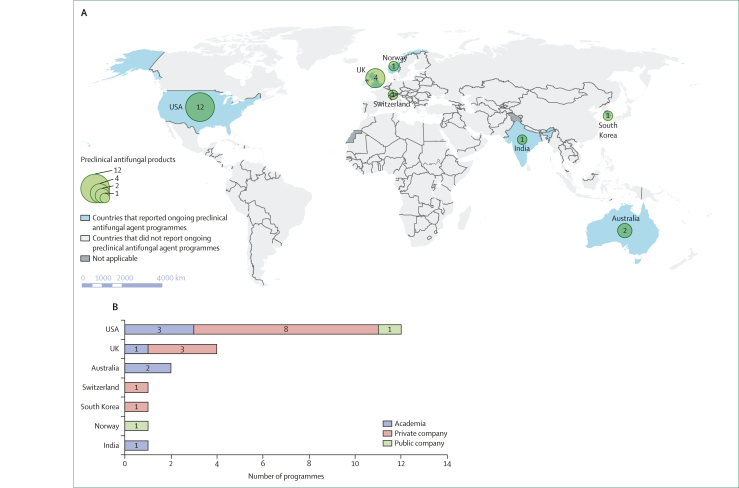
Distribution of preclinical antifungal programmes by geography and institutions (A) World map showing the distribution of antifungal programmes.^2^ (B) Breakdown of antifungal programmes by country and institution.

Although the scope of the analysis was global, programmes were distributed across seven countries (figure 3A), primarily in Europe and North America. According to the 2024 survey, more than half of the programmes were from the USA (12 [55%] of 22), followed by the UK (four [18·1%] of 22), Australia (two [9%] of 22), and a few others (figure 3B). All programmes, except one (21 [95·5%] of 22), which was based in India, were based in high-income countries. These observations are at odds with the evidence that low-income and middle-income countries bear the greatest burden of fungal infections.^1^

### Highlights of novel agents in the preclinical pipeline

Of the 16 new compounds, eight were in advanced stages: three were in IND-enabling studies and five were preclinical candidates. The other eight programmes that involved new compounds were at the lead-optimisation stage and included one programme in which systemically available analogues of the topical compound ATB1651 were being evaluated and are currently being advanced in phase 2 for the treatment of individuals with mild to moderate onychomycosis (table).

Of the three programmes involving new compounds that were in IND-enabling studies, two have progressed to the clinical stage since the closing date for the WHO antifungal pipeline report.^2^ SCY-247, being developed by SCYNEXIS (Jersey City, NJ, USA) to treat IFIs, is a second-generation fungerp compound that interferes with the synthesis of the fungal cell wall polymer β-(1,3)-d-glucan; the phase 1 clinical trial of SCY-247 was initiated in mid-December, 2024.^12^ This compound has broad antifungal activity against *Candida* spp and *A fumigatus* and potentially offers dosing flexibility with both intravenous and oral formulations.^13^ Occidiofungin (as the OCF001 gel formulation), being developed by Sano Chemicals (Bryan, TX, USA) to treat recurrent vulvovaginal candidiasis, completed a phase 1 clinical trial in December, 2024; the phase 1 study showed that the compound was safe and well tolerated.^14^^,^^15^ Occidiofungin is a cyclic 8-amino-acid residue peptide with broad activity against *Candida* spp. The other product in the IND-enabling stage is NP339, a broad membrane-acting fungicidal peptide being developed for invasive and respiratory fungal infections. Novabiotics (Aberdeen, UK), the developer of NP339, lists both intravenous and inhaled formulations, which would introduce additional flexibility in clinical treatment, if successful.^16^

Five programmes involving new compounds were listed as having preclinical drug candidates. Vitalex Biosciences (Torrance, CA, USA) is developing VX01, which is a humanised monoclonal antibody that targets the CotH proteins encoded by Mucorales fungi.^17^^,^^18^ Mucorales infections, or mucormycosis, are severe infections that typically occur in individuals who are immunocompromised, such as people undergoing chemotherapy or receiving stem cell transplants.^19^ Brigid Biologics is a new biotechnology company that was recently spun out from the University of Aberdeen (Aberdeen, UK) and is developing a pan-*Candida* spp monoclonal antibody (indicated as next-generation antifungal antibodies in table) to treat invasive candidiasis. Two related programmes from the University of Texas MD Anderson Cancer Center (Houston, TX, USA) were developing bioengineered cell-based biological agents. These programmes used chimeric antigen receptor (CAR) engineering to produce human CAR T cells directed against either clinically relevant *Aspergillus* or *Cryptococcus* species.^20^, ^21^, ^22^

The only small-molecule candidate in advanced stage was a small-molecule inhibitor targeting specific fungal inositol polyphosphate kinases. This compound, being developed at the Westmead Institute for Medical Research and University of Sydney (Sydney, NSW, Australia), targeted *C neoformans* and *Candida* spp (shown as DT-3-318 series in the table).^23^ This inhibitor class blocks the synthesis of inositol polyphosphate, which is a small molecule at the interface of cell signalling and energy metabolism and is crucial for fungal virulence. The fungal and human inositol polyphosphate kinase mechanisms differ, with specific redundancies being absent in the fungal pathway. Thus, this distinctive and novel fungal target could represent a novel approach in tackling IFIs while mitigating toxicity, which is a common challenge in antifungal therapies that target mechanisms shared with human homologues.

Four advanced-stage programmes were exploring new formulations of known small-molecule antifungal compounds, either approved or in clinical development. Two of these programmes, investigating an inhaled dry-powder formulation of opelconazole (Pulmocide, London, UK) and both oral and inhaled formulations of BSG005 (Biosergen AS, Tiller, Trondheim, Norway), were in the IND-enabling stage. The remaining two programmes were in the preclinical candidate stage and included intravenous and inhaled formulations of olorofim (F2G Therapeutics, Macclesfield, UK) and a novel liposomal formulation of antifungal drugs being developed at the University of Georgia (Athens, GA, USA). The novel liposomal formulation represents a new drug delivery formulation consisting of DectiSomes, which are liposomes coated with Dectin-2, a mammalian C-type lectin receptor. These DectiSomes specifically target fungal pathogens, help in mitigating toxicity, and enhance the activity of the encapsulated antifungal agent amphotericin B.^24^ Existing antifungal classes, such as azoles, echinocandins, and polyenes, primarily act on well established fungal mechanisms: inhibition of ergosterol synthesis, interference with β-(1,3)-D-glucan cell wall synthesis, and disruption of membrane permeability.

### Company size

Seven (32%) of 22 programmes were conducted in academic institutions (one specific university in the USA had two unique programmes), whereas only two (9%) were from publicly funded companies (figure 2B); among these companies, one company was classified as micro (fewer than ten employees) and the other as small (11–50 employees). Ten different privately funded companies conducted the remaining 13 programmes (nine each conducted a single programme, and one specific company conducted four programmes). All ten of these biotech companies had fewer than 50 employees, and five of them had fewer than ten employees. Overall, the preclinical antifungal pipeline relied almost entirely on micro (fewer than ten employees; n=6) and small (≤50 employees; n=6) commercial entities, along with academic institutions (n=6), to advance innovative antifungal treatments. This distribution considerably increases the developmental risk for these products.

This pattern is also indicative of the number of large pharmaceutical companies that have exited the antibacterial and antifungal discovery areas, as the same trend was observed for both preclinical and clinical antibacterial programmes.^25^^,^^26^ Furthermore, the fact that approximately a third of the antifungal programmes stem from academic institutions (n=6) highlights further risk in the preclinical antifungal pipeline, as these academic institutions often do not have access to the required experience for dealing with advanced aspects of drug development, knowledge of the broader ecosystem, including potential funders, and might require partnering. Thus, the heavy reliance on small and academic entities contributes to the overall vulnerability and instability of the pipeline in developing innovative antifungal treatments.

## Discussion

The preclinical pipeline is thin and fragile, with only 22 programmes from 18 developers active in this space. Most of the programmes focused on direct-acting molecules, whereas the remaining programmes investigated antibody-based biological products. For all FPPs, although specific agents with some proof of activity were available in the preclinical pipeline, the critical-priority pathogens had a considerably higher number of targeting products than the pathogens of other groups. Nine programmes included compounds that showed activity against all four members of the critical-priority pathogen group, with seven of them being new compounds; for the remaining two compounds, a new administration route was being investigated.

Although we performed a global review, development was exclusive to seven countries, mostly in North America and the European region, calling for an equitable development aligned with the disease burden. When contextualised against the antibacterial preclinical pipeline, the antifungal pipeline shows both shared and unique challenges. As with antibacterials, antifungal agents face high attrition during early development, with 9 (41%) of 22 programmes still at the lead-optimisation stage and only 5 (23%) of 22 at the IND-enabling stage. The antifungal pipeline heavily relies on small biotech firms and academic institutions, with more than 80% of developers employing fewer than 50 employees. This pattern mirrors the fragmentation seen in antibacterial R&D.^26^ However, the antifungal pipeline is substantially smaller and less mature than the 2025 WHO antibacterial pipeline, which included 232 preclinical agents targeting priority bacterial pathogens.^27^ Furthermore, compared with past antifungal pipelines, the 2024 pipeline shows only a modest increase in the number of programmes. For example, the 2022 preclinical pipeline included 18 candidates, whereas the current analysis identified 22. Although non-traditional approaches (eg, bacteriophages and anti-virulence agents) are increasingly being used for the development of antibacterial agents, antifungal innovation is still dominated by small molecules, with only a few biological or immunomodulatory strategies.

Antifungal innovation is constrained by the low number of fungal-specific targets and the complexity of fungal biology, both of which necessitate increased investment in basic research. Finally, low awareness about IFIs and antifungal resistance constrains investment in this area. This challenge is compounded by geopolitical shifts and funding uncertainties in countries where preclinical antifungal programmes are concentrated. Budgetary constraints affecting major public research institutions might substantially affect the sustainability and progression of antifungal R&D.

Although not part of the present Review, univalent or multivalent vaccines represent a valuable complement to therapeutic approaches, particularly for preventing fungal infections in vulnerable and high-risk populations; hence, the development of such vaccines should also be encouraged.^28^

This Review has some limitations. First, both data analysis and activity assessment were based on self-declared information from antifungal drug developers. This reliance on self-reported data is a limitation of our review process, as some programmes might include compounds with anti-FPP activity that is not being tested in the initial developmental stage. Second, the activity was declared based on in-vitro studies or in-vivo animal models, and additional evidence regarding the efficacy of these compounds in humans will be needed when they enter clinical development.

Although our analysis excluded vaccine programmes, several programmes are underway with both univalent and multivalent antifungal vaccines progressing towards clinical development and registration. Development of potential vaccines complements the prospect of prevention of fungal infections, which would be valuable in susceptible and high-risk populations. Combination therapy with different antifungal agents should also be investigated as a strategy to address resistant infections.

## Conclusions

This Review presents an overview of the WHO global analysis of publicly available preclinical antifungal pipeline projects and shows how alarmingly low the level of therapeutic preparedness is to curb the rising burden of IFIs and antifungal resistance. IFIs caused by FPPs represent a serious and mounting public health concern. However, there are insufficient preclinical programmes to deliver a robust pool of clinical candidates to address the evolving landscape of these infections. In addition, the existing pipeline is fragile owing to the small size and capacity of the developers’ group. Additional efforts and investments are needed to ensure that effective treatments against FPPs can be developed in the next 15–20 years. A strong need exists for increased R&D efforts, driven by an urgent and focused intention to tackle this important yet overlooked area of infectious diseases.

## Declaration of interests

RAA and TR are consultants to the WHO Antimicrobial Resistance Department. All other authors declare no competing interests.
